# A longitudinal study of changes in interactional justice and subsequent short-term sickness absence among municipal employees

**DOI:** 10.5271/sjweh.3927

**Published:** 2021-03-01

**Authors:** Mika Koskenvuori, Olli Pietiläinen, Marko Elovainio, Ossi Rahkonen, Aino Salonsalmi

**Affiliations:** Department of Public Health, University of Helsinki, Helsinki, Finland; Department of Psychology and Logopedics, University of Helsinki, Helsinki, Finland

**Keywords:** between-individual, change-effect, fixed effect, organizational justice, self-certified sickness absence, sickness absence spell, within-individual

## Abstract

**Objectives:**

Level of perceived interactional justice has been shown to be associated with sickness absence, but less is known about the effects of changes in interactional justice. It is also unknown to what extent unmeasured, time-invariant differences contribute to the association. We investigated the association between interactional justice changes and subsequent short-term (1–3 days) sickness absences over a 12-year follow-up using between- and within-individual modeling among ageing municipal employees.

**Methods:**

The data was derived from Helsinki Health Study cohort with baseline survey in 2000–2002 (N=8960, response rate 67%) and follow-up surveys in 2007 and 2012 (response rates 79% and 83%, respectively). At baseline, participants were 40–60-year-old employees of the City of Helsinki, Finland. Sickness absences from the employer’s registry were linked with the responses (78%). The analytic sample was 2109 and 2070 individuals for between-individual and 4433 individuals and 8425 observations for within-individual associations.

**Results:**

Negative change in interactional justice was associated with an increased risk of short-term sickness absence in between-individual models after adjusting for age and gender. Adjustment for sickness absence history attenuated the association. In within-individual models, a negative change in perceived interactional justice was associated with an increased risk of short-term sickness absence spells [incidence rate ratios (IRR) 1.05 (95% confidence interval 1.01–1.09)]. This association was robust to adjustments for gender, age, health behaviors and sickness absence history.

**Conclusions:**

Paying attention to management principles – especially managerial behavior and treatment of employees to avoid the deterioration of the level of interactional justice – may provide a way of reducing self-certified short-term sickness absence spells.

One established theoretical model explaining the association between work-related psychosocial factors and employee health is the organizational justice model of occupational strain (1). The model is based on the idea that, in addition to distributing resources and obligations within organizations, also the procedures and rules that guide decision-making in organizations matter. Studies of these rules and procedures have provided the basis for a new line of research examining decision-making and social relationships in working communities, ie, distributive, procedural and interactional justice that are the basic dimension of organizational justice. The idea behind the interactional justice dimension (2) suggests that people are not only sensitive to the actual decisions or the procedures leading to decisions, but also the way they are treated by those making the decisions. It has been argued that in today’s increasingly social work life, the interactional dimension of organizational justice becomes increasingly important to employee wellbeing and health (3).

Unfair treatment has been shown to evoke strong negative emotions, such as anger, anxiety and psychological distress (4), that in turn have direct physiological links to adverse health outcomes. Perceived injustice has been associated with vascular dysfunction (5), metabolic syndrome (6) and inflammatory markers (7). Indeed, the levels of various dimensions of organizational justice have repeatedly been shown to be associated with various health outcomes, including protection from sickness absence following a major life event (8), sickness absences among older employees (9) or among employees with mental disorders symptoms (10–11), disability pension (12) and retirement intentions (13). Specifically, the association between the interactional justice dimension and employee health have shown to be strong (3, 14).

In previous studies, the potential effects of organizational justice perceptions have been studied by calculating levels of perceived justice at two time points and comparing individuals with constantly low to those with increasing, decreasing or constantly high perceived organizational justice. The analyses have been conducted by comparing the effects between individuals reporting low organizational justice to the individuals reporting high organizational justice (so called between-individual analysis). This methodology has two problems: (i) the cut-off points for high and low groups may be artificial and the cut-off point may change over the years and ii) the between-individual analysis is unable to consider confounding by all time-invarying confounders (the unmeasured differences between the groups that explain the relationship between the organization justice and the outcome variable) (15). This effect can be mitigated by using the so called within-individual analysis, where each individual acts as a reference only to themselves, effectively removing the effects of unmeasured time-invariant differences between the individuals.

Other problems in previous studies include monomethod bias (measuring both exposure and outcome using the same measure) and short follow-ups. In this study, we used register-based sickness absences as our health indicators and examined the association between the changes in interactional organizational justice and short-term sickness absence spells (SSAS) over 12-year follow-up and three measurement points. We used both between and within-individual modeling among ageing employees of the capital city of Finland, Helsinki, to find out possible association between changes in interactional justice and SSAS and compare two different modeling modalities.

Sickness absences are often classified according to their length – to short- and long-term spells – or according to the certification method – to self- or medically certified. Reasons for short- and long-term absences are thus somewhat different. SSAS (1–3 days) are often self-certified and thus do not require a medical verification – therefore short absences may be less closely related to medically certified conditions. SSAS are more likely than medically certified sickness absence spells to reflect problems in working conditions, of which interactional justice is a significant component (12).We presumed that within-individual perceived association between organizational justice and SSAS is smaller when compared to between-individual perceived one, but that the association will be more robust with respect to a number of confounders.

## Methods

### Data

This study is part of the Helsinki Health Study examining the health and well-being of the ageing employees of the City of Helsinki (16). The City of Helsinki is the largest employer in Finland with approximately 40 000 employees, 72% of whom are women (17). The range of occupations is large and heterogeneous with over 100 occupational titles, covering both blue- and white-collar jobs. The data consisted of the Helsinki Health Study baseline (2000–2002) and two follow-up (2007, 2012) questionnaire surveys and the City of Helsinki personnel register data on sickness absence. All employees reaching 40, 45, 50, 55, and 60 years in 2000, 2001, and 2002 received the baseline survey. Altogether, 8960 employees responded to the baseline survey (response rate: 67%), 7332 to the first follow-up survey (response rate: 83%) and 6809 to the second (response rate: 79%). The follow-up questionnaires were only sent to the individuals who responded to the baseline questionnaire. As the baseline data were collected over three consecutive years, the period between baseline and follow-up surveys varies between 5–7 years. However, the first questionnaire in 2000 (N=3141) did not contain the questions about organizational justice, which limited the study population. The survey data were linked to the City of Helsinki personnel register data on sickness absence for those with written consent for such linkage (78%, N=5893). To be included in the study population in the between-individual analysis, a person must have responded to the baseline (2001–2002) and at least one follow-up (2007 or 2012) survey.

Individuals were excluded from the study from the moment they were no longer employed by the City of Helsinki. The largest reason for the attrition was retirement (N=1272 and 2210 in 2007 and 2012 respectively). In addition, those with missing values in any of the study variables were excluded from the between-individual analyses to assure the comparability of different models. However, they were not excluded from the within-individual analysis if they had any complete observations in any of the survey waves. No inputation was used for any data.

The final study population in the between-individual analysis comprised of 2109 and 2070 individuals for the baseline–2007 wave and 2007–2012 wave, respectively and 4433 individuals and a total of 8425 observations for the within-individual analysis. The gender split in the study reflects well the typical gender split in Finnish public sector (80% women).

The Ethics Committees of the Department of Public Health, University of Helsinki, and the health authorities of the City of Helsinki approved the study.

### Measures

*Interactional justice*. The level of interactional justice was calculated using the four questions included into the Helsinki Health Study from the original six questions introduced by Moorman (18): (i) Our superior listens to the viewpoints of the employees in important subjects; (ii) Our superior is able to suppress personal bias; (iii) Our superior treats the employees with kindness and consideration; and (iv) I can trust our superior (Cronbach’s α = 0.89, 0.89 and 0.91 in baseline, 1^st^ follow-up and 2^nd^ follow-up, respectively). Each question had five different alternative answers from fully object (value 1) to fully agree (value 5). A sum score (4…20) was calculated from this for each participant at each wave.

The interactional justice score was dichotomized at lowest tertile on the baseline. For the between-individual analysis the participants were split into four different categories according to the changes in the reported interactional justice: (i) stable high, (ii) stable low, (iii) change low-high and (iv) change high-low. The change was determined based on the results of two consecutive waves (baseline – >1^st^ follow-up and 1^st^ follow-up – >2^nd^ follow-up).

For the within-individual analysis, the same dichotomy for interactional justice was used to define each individual’s perception of interactional justice at maximum of three time points (baseline, 2007 and 2012). Here the individuals were no longer grouped according to the change in perceived interactional justice, but the change was introduced at each time point by comparing the measured value of interactional justice with the mean value of interactional justice for each individual (see statistical methods). It should be noted that no universally agreed cut-off point exists for dichotomization, and a hybrid model would allow alternative methods for the treatment of interactional justice. However, in order to maintain the comparability between the models used in this study and with the previous works, a dichotomization approach was chosen.

### Sickness absences

For Finnish municipal employees, SSAS are employee self-certified, 1–3-day absences. Longer absences require medical confirmation. The amount of SSAS for each individual was obtained from the employer’s register, however, the causes of SSAS’s were not available. The amount of SSAS were calculated for the three-year period following the first and second follow-up questionnaires or until the person was not any more employed by the City of Helsinki due to changing jobs or retirement. The average follow-up times were 2.7 and 2.6 years after the first and second follow-up questionnaires, respectively. The amount of SSAS during each three-year period following the measurement of interactional justice was used as a dependent (=outcome) variable in all the models.

Long-term sickness absences were not controlled as a separate covariate, but their duration as well as duration of other absences from work was deduced from the amount of working days to reduce the exposure time (see statistical methods).

### Covariates

Gender and age were measured at baseline and used as covariates in all the models. Because no gender interactions (P>0.1) were found, men and women were analyzed together. As previous sickness absence is a significant predictor for the sickness absences (19), the amount of SSAS during the baseline survey (2000–2002) was added as a covariate. The rest of the covariates were measured simultaneously with the measurement of the perceived interactional justice in each wave (ie, the covariates were time variant). The treatment of the covariates was different for between- and within-individual analyses (see statistical methods).

Being overweight and health behaviors were assessed via dichotomous variables: overweight (self-reported height / weight with cut-off at BMI >25 kg/m^2^) and three variables representing health behaviors: alcohol use (problem drinker on CAGE questionnaire) (20), tobacco smoking (after individual responses for current smoking) and leisure-time physical activity [total of four questions on physical activity were converted to approximate metabolic equivalent (MET) index with cut-off at 14 MET hours] (21).

All the models in within-individual analysis were adjusted with additional random effect parameter for survey wave to take into account potential differences due to the measurement phase, workplace and sickness absence compensation. Table 1 describes the study population in detail.

**Table 1:** The study population. [MET=metabolic equivalent; obs=observations; SSAS=short-term sickness absence spells.]

	Between-individual Baseline–2007 (N=2109)		Between-individual 2007–2012 (N=2070)		Hybrid model: within and between-individual (Baseline–2012) Individuals (N=4433) Observations (N=8425)			
		
	N	%	N	%	N	%	Obs	%
Total number of obs	2109		2070		8425			
Mean number of obs	1		1		1.90			
Number of SSAS	11 787		10 353		45 389			
Number of working days	1 746 390		1 649 917		7 066 434			
Number of SSAS / 100 working days	0.67		0.63		0.64			
Men	340	16	329	16	782	18		
Women	1769	84	1741	84	3651	82		
Interactional justice								
Stable high	1055	50	1024	49	N/A			
Stable low	349	17	366	18	N/A			
Change low-high	342	16	344	17	N/A			
Change high-low	363	17	336	16	N/A			
Problem drinker	499	24	642	31			2040	24
Non-problem drinker	1610	76	1428	69			6385	76
Smoker	373	18	324	16			1602	19
Non-smoker	1736	82	1746	84			6823	81
Overweight	1062	50	1141	55			4164	49
Normal weight	1047	50	929	45			4261	51
MET inactive	435	21	497	24			1920	23
MET active	1674	79	1573	76			6505	77

### Statistical methods

Due to the overdispersion of the data, negative binomial regression was used to calculate the incidence rate ratios (IRR) and their 95% confidence intervals (CI). Because the amount of working days during the study period varied among the study population, the absolute amount of SSAS was transformed to rate data (number of SSAS per working days) by adding the natural logarithm of the working days during the observation periods as an offset-parameter to the models. Therefore, the amount of working days represented the amount of exposure to SSAS for each individual. Three different models were used in both phases of modeling with varying covariates: model 1 = gender and age of the individuals at baseline, model 2 = model 1 + baseline SSAS, and model 3 = model 2 + weight and health behaviors. The analysis was done in two parts: between- and within-individual association.

### Between-individual association

First the between-individual association between the change in interactional justice and SSAS was studied using generalized linear model. The reference category was interactional justice stable high to which the association between the change in interactional justice and risk for SSAS in other groups was compared.

### Within-individual association

To further analyze the association between changes in individual’s experience of interactional justice and SSAS and remove the effect of unobserved bias from the results, the association between the changes in interactional justice and SSAS was studied using within-individual (or fixed-effects) regression analysis. Here the data from all three waves was pooled into a common pool of observations and used to derive within-individual estimates for the association between the interactional justice and SSAS. The within-individual analysis automatically controls for all the obvious time-invariant variables, such as gender and age at baseline, but also for all the other unmeasured time-invariant personal, demographic or environmental factors that may cause bias in the results (22). Disregarding the between-association and focusing only on within-association is also an effective method to study if the effects of *change* in outcome variable are associated with *changes* in the explanatory variable (23).

The within-individual analysis is typically performed using a model with random effects. Random effects model assists in controlling the unobserved heterogeneity by splitting the unexplained variance into different levels of hierarchy (in this study temporal and individual levels) by introducing a residual (ie, error term) at each level. This splitting also allows the model to treat the lower-level entities unidentical thus maintaining for example the temporal hierarchy and giving the opportunity to make use of the longitudinal nature of the data (24).

However, conventional random effects model assumes that the residuals are uncorrelated with the observed covariates, which is typically an unrealistic assumption. If this assumption is violated, the model coefficients are biased. Another common way to introduce the individual specificity into a model is to use fixed-effects modeling. This can be achieved for example by introducing a dummy variable corresponding each individual to the model or typically more computationally efficiently by replacing the values of the covariates at each wave with the individual variances (deviation from each individual’s mean score for each variable). However, it has been shown that conditional fixed effects do not truly control for the fixed covariates when used with negative binomial regression models (25).

To overcome the limitations of both models, an alternative solution, a so-called hybrid model, was selected as proposed by Allison (26) in the spirit of Mundlak (27) and previously used elsewhere (28–30). In the hybrid model, a random effects model is fitted with two variables for each time-varying regressor: individual-specific mean value and individual-specific variance to control for the between- and within-individual effects, respectively. This is done for all the models of hybrid-model modality by adding the individual specific mean and variance values as regressors. The hybrid model used gives the possibility to examine both within- and between-individual associations simultaneously and the credibility of hybrid model can be improved by comparing the associations for both dependent variables and covariates from traditional between-individual to associations decomposed from the hybrid model.

All the analyses were done using R (31) and the hybrid-model analysis used the *glmmTMB*-package (32).

## Results

Table 2 shows the mean amount of SSAS for employees with exposures to different changes in interactional justice. The employees experiencing constant high interactional justice (stable high) had the smallest amount of SSAS as such (5.25) and when compared to the working days (0.63 per 100 working days). The negative change group (change high-low) had the highest amount of SSAS (6.14) and most days (0.75 / 100 working days). The mean amount of SSAS decreased from 1^st^ to 2^nd^ follow-up.

**Table 2:** Amount of short-term sickness absence spells (SSAS) in groups experiencing different changes in interactional justice.

Interactional justice change	Baseline (3 years)		1^st^ follow-up (average length 2.7 years)		2^nd^ follow-up (average length 2.6 years)	
		
	Mean SSAS / person (amount)	SSAS / 100 working days	Mean SSAS / person (amount)	SSAS / 100 working days	Mean SSAS / person (amount)	SSAS / 100 working days
All	5.59	0.61	5.59	0.67	5.00	0.63
Stable high			5.25	0.63	4.74	0.59
Stable low			5.95	0.71	5.19	0.67
Change low-high			5.69	0.69	4.92	0.61
Change high-low			6.14	0.75	5.67	0.72

### Between-individual association

Table 3 summarizes the IRR values and 95% CI of the between-individual analyses. For all the models and both waves, the individuals experiencing constant high interactional justice (stable high) had the lowest risk for SSAS. Being younger or of female gender increased the risk. In the first model with only gender and age as covariates, the risk was increased by 12% (15% in 2007–2012 wave) or 16% (16% in 2007–2012 wave) when the individual was exposed to stable low or change to low interactional justice, respectively. Association for change to high interactional justice was not statistically significant. Adjusting the model for baseline SSAS attenuated the associations, and only interactional justice change to low in 2007–2012 data set remained at 13% increased risk (95% CI 1.02–1.26).

**Table 3:** Incidence rate ratios (IRR) and their 95% confidence intervals (CI) of the association between changes in interactional justice and short-term sickness absence spells (SSAS), generalized linear model - negative binomial regression – between-individual association. [MET=metabolic equivalent].

	Model 1 ^a^		Model 2 ^b^		Model 3 ^c^	
		
	IRR	95% CI	IRR	95% CI	IRR	95% CI
Baseline-2007						
Interactional justice						
Stable high (reference)	1.00		1.00		1.00	
Stable low	1.12	1.00–1.24	1.04	0.94–1.14	1.02	0.93–1.13
Change low-high	1.07	0.96–1.19	0.97	0.87–1.07	0.95	0.86–1.05
Change high-low	1.16	1.05–1.29	1.10	1.00–1.21	1.10	1.00–1.21
Age	0.97	0.97–0.98	0.98	0.98–0.99	1.02	1.01–1.02
Gender (reference: male)	1.72	1.52–1.94	1.35	1.21–1.49	1.44	1.29–1.60
Baseline SSAS			1.10	1.04–1.16	1.09	1.09–1.10
Overweight					1.19	1.10–1.27
Smoking					1.11	1.02–1.22
Alcohol					1.13	1.04–1.22
Low MET					0.98	0.89–1.06
2007–2012						
Interactional justice						
Stable high (reference)	1.00		1.00		1.00	
Stable low	1.15	1.02–1.30	1.05	0.94–1.16	1.04	0.94–1.16
Change low-high	1.05	0.93–1.19	0.98	0.88–1.10	0.98	0.88–1.10
Change high-low	1.16	1.03–1.31	1.14	1.02–1.27	1.13	1.02–1.26
Age	0.98	0.97–0.99	0.99	0.99–1.00	0.99	0.99–1.00
Gender (reference: male)	1.76	1.54–2.00	1.46	1.31–1.63	1.53	1.36–1.71
Baseline SSAS			1.09	1.09–1.10	1.09	1.08–1.10
Overweight					1.14	1.05–1.23
Smoking					1.08	0.97–1.20
Alcohol					1.06	0.98–1.15
Low MET					1.05	0.96–1.15

a Adjusted for gender + age.

b Adjusted for Model 1 + baseline SSAS.

c Adjusted for Model 2 + overweight and health behaviors. Health behaviors consisted of problem drinking, tobacco smoking and leisure-time physical activity.

In general, the risk for SSAS among individuals experiencing a positive change in interactional justice (change low-high) was lower than for the individuals experiencing either a negative change or constant low interactional justice. It can also be observed on both waves that individuals experiencing a change to low interactional justice had a greater risk for SSAS than individuals exposed to constant low interactional justice. However, when adjusting for baseline SSAS (model 2) and health behaviors (model 3) the associations attenuated (IRR 1.10, 95% CI 1.00–1.21)

### Within- and between individual associations from the hybrid model

Table 4 summarizes the IRR values for both within- and between-individual associations decomposed from the hybrid model when the perceived interactional justice changes from high to low. For within-individual association, the change in interactional justice from high to low was associated with IRR 1.05 (95% CI 1.01–1.09) fold increase in the risk of SSAS, and this association was robust for adjustment for various variables. In between-individual analysis the change was associated with IRR 1.19 (95% CI 1.10–1.29) fold increase in the risk of SSAS in model 1 and was not statistically significant once the model was adjusted for baseline SSAS (model 2) and overweight and health behaviors (model 3).

**Table 4:** Results from hybrid model describing the incidence rate ratios (IRR) of short-term sickness absence spells (SSAS) as a response to change in interactional justice (IJ) from high to low. [MET=metabolic equivalent.]

	Model 1 ^a^		Model 2 ^b^		Model 3 ^c^	
		
	IRR	95% CI	IRR	95% CI	IRR	95% CI
IJ – within association	1.05	1.01–1.10	1.05	1.01–1.09	1.05	1.01–1.09
IJ – between association	1.19	1.10–1.28	1.04	0.98–1.11	1.03	0.97–1.09
Gender (ref: male)	1.95	1.80–2.11	1.47	1.38–1.56	1.55	1.45–1.65
Age	0.98	0.97–0.98	0.98	0.98–0.99	0.98	0.98–0.99
Baseline SSAS			1.09	1.09–1.10	1.09	1.09–1.09
Alcohol – within association					0.99	0.94–1.05
Alcohol – between association					1.10	1.04–1.17
Smoking – within association					0.92	0.85–0.99
Smoking – between association					1.14	1.07–1.21
Overweight – within association					1.00	0.94–1.06
Overweight – between association					1.21	1.15–1.27
Low MET – within association					0.97	0.93–1.01
Low MET – between association					0.99	0.92–1.06

a Model 1 = Gender + age.

b Model 2 = model 1 + baseline SSAS.

c Model 3 = model 2 + overweight and health behaviors. Health behaviors consisted of problem drinking, tobacco smoking and leisure-time physical activity.

## Discussion

The aim of the study was to assess the association between changes in interactional justice and SSAS among municipal employees using two methodologies: between- and within-individual. Both modelling methodologies indicated a small association between negative change in interactional justice and SSAS, but the magnitude of the association was weaker in within-individual models. Also, the contribution of SSAS history and health-related covariates was examined. Adjustment for SSAS history attenuated the association in between-individual models whereas the association remained after all adjustments in within-individual models.

Previous studies of the association between interactional justice and sickness absence are scarce. A study by Hjarsbech et al (10) on 1034 Danish employees found no association between levels of interactional justice and long-term sickness absences. Elovainio et al (8) showed that despite the perception of interactional justice, employees had as much sickness absences from work after a major life event, but they returned to work after a longer period if they perceived interactional justice to be low, compared to those who perceived interactional justice to be medium or high. In a study by Head et al (33) on British civil servants, the risk for short-term sickness absence was 4–26% higher for individuals experiencing low interactional justice depending on the model and study population. In study by Spanier et al (34), the risk for self-reported sickness absences was 34% higher for the group that experienced low organizational injustice. Even though none of the studies analyzed the association between changes of interactional justice and SSAS, the results are comparable to this study. The previous studies did not, however, adjust for the baseline SSAS and utilized between-individual modelling. The magnitudes of the associated risks are of the same order as for risks associated with increase in job demand or decreased job control (35–37), which have been reported to be in the order of 10–40%.

In this study, the magnitude of the between individual associations for dependent variables and confounders were similar in both the traditional between-individual modeling using generalized linear model and between-individual association decomposed from the hybrid model. Still, the magnitude of association differed from within-individual modelling suggesting unobserved time-variant differences between the individuals exist that play role in the association. Thus, ignoring unobservable differences might overstate the association. In addition, between-individual modeling was not able to capture statistically significant association between the changes in interactional justice and SSAS after adjustment for SSAS history. Our results thus suggest that within-individual model might be a better approach to study the association between changes in perceptions of justice and SSAS.

The low magnitude of the association in within-individual analysis (5%) is not a surprise as major contributors to sickness absence are typically health-related. This reasoning is further supported by the finding that the between-individual association is higher than within-individual association. In practice, it insinuates that individuals who report low organizational justice during some study interval are prone to having higher levels of SSAS also during the periods when they do not report low organizational justice. The analysis of reasons for this are beyond the scope of this article, but health-related factors cannot be excluded. Further fortification can be drawn from the observation of decreased association when health-covariates are adjusted. The modest magnitude of the association is also in line with the arguments by Zapf et al (38) that small correlations are expected in longitudinal studies as physical and mental health are influenced by a multitude of factors.

In the within-individual model, the associations were found to be robust for adjustments for gender, age, SSAS history, weight and health behaviors. The results thus suggest that negative change in interactional justice truly contributed to the association instead of only reflecting the contribution of previous sickness absence spells and weight and health behaviors. In addition to the covariates described in the text, the effects of common mental disorders (after GHQ-12 questionnaire), shift work and mentally and physically strenuous job conditions were also examined, but they did not affect the association (data not shown).

The overall decline in the amount of sickness absences with respect to study waves (table 2) gives a hint about the signs of “healthy-worker effect” (HWE) in the cohort, where the healthier employees stay longer in the working population thus effectively lowering the mean amount of sickness absences with respect to time (39). As the HWE depends on time, it is not corrected for in within-individual analysis. On the other hand, the work ability should be seen as a continuum or multidimensional rather than a binomial variable (40). This way the effect of proper organizational justice can even increase sickness absence spells, while on the other hand prolonging the working career and delaying the retirement due to work disability. This analysis is, however, outside the scope of this study.

A clear strength of the study is the use of within-individual analysis to complement the between-individual analysis. The study benefits from follow-up data on employed people combined with a register-based data on sickness absence which increases the credibility on the data.

However, data from only one public sector employer was used and thus a question arises whether the results can be generalized also to private sector or to industry. Also due to the typical feature of Finnish municipal employers, the women are significantly more represented than men. Thus, the data cannot directly be generalized to all the employees but has a potential to be generalized to municipal employees or public sector employees. Also, even though the data contains baseline and two follow-up studies, the amount of data points for each individual in within-individual analysis is modest at best. Therefore, future studies should include more study waves to increase the number of data points as more follow-up studies become available. Increasing the number of data points would open the possibility to solve the problems related with the dichotomization of the predictor variable as with the hybrid model the interactional justice can be treated as a continuous predictor. Additionally, with a continuous predictor, also curvilinear (eg, quadratic) relationships with the outcome variable could be explored. The future studies should also aim for populations with more men and for the private or industrial sector employees.

### Concluding remarks

In conclusion, the study showed that a negative change in interactional justice was associated with the risk of SSAS. In within-individual analysis, this association was robust against a number of confounders, including SSAS history, suggesting true contribution of interactional justice. In future studies, within-individual modeling might have an advantage to between-individual analysis that has typically been used.

The associated risk between the change in interactional justice and SSAS was rather small but as the association is established, paying attention to improving the interactional justice might aid in reducing SSAS without implying major costs to the employer.
